# Leg Prosthesis With Somatosensory Feedback Reduces Phantom Limb Pain and Increases Functionality

**DOI:** 10.3389/fneur.2018.00270

**Published:** 2018-04-26

**Authors:** Caroline Dietrich, Sandra Nehrdich, Sandra Seifert, Kathrin R. Blume, Wolfgang H. R. Miltner, Gunther O. Hofmann, Thomas Weiss

**Affiliations:** ^1^Department of Clinical Psychology, Friedrich Schiller University, Jena, Germany; ^2^Berufsgenossenschaftliche Kliniken Bergmannstrost Halle/Saale, Halle, Germany; ^3^Department of Trauma, Hand and Reconstructive Surgery, University Hospital Jena, Jena, Germany

**Keywords:** somatosensory feedback, prosthesis, lower leg amputation, phantom limb pain, functionality, prosthesis training

## Abstract

Phantom limb pain (PLP) develops in most patients with lower limb amputation. Changes in the peripheral and central nervous system (CNS) are hypothesized to contribute to PLP. Based on ideas to modify neural reorganization within the CNS, the aim of the study was to test, whether prostheses with somatosensory feedback might help to reduce PLP, and increase the functionality of movement with a prosthesis. We therefore equipped the prostheses of 14 lower leg amputees with a simple to use feedback system that provides electrocutaneous feedback to patients’ thigh whenever the foot and toes of the prosthesis touch the ground. Two weeks of training with such a feedback prosthesis reduced PLP, increased the functional use of the prosthesis, and increased patients’ satisfaction with prosthesis use. We found a significant overall reduction of PLP during the course of the training period. Most patients reported lower PLP intensities at the end of the day while before training they have usually experienced maximal PLP intensities. Furthermore, patients also reported larger walking distances and more stable walking and better posture control while walking on and across a bumpy or soft ground. After training, the majority of participants (9/14) preferred such a feedback system over no feedback. This study extends former observations of a similar training procedure with arm amputees who used a similar feedback training to improve the functionality of an arm prosthesis in manipulating and grasping objects.

## Introduction

Major amputations of the lower limb are more prevalent than amputations of the upper limb (1). Approximately 84% of people affected by amputation wear a lower limb prosthesis (2) for walking and other purposes of daily living. Common lower limb prostheses support walking, bending the knee joint, and absorb shocks and stabilize stance. However, they lack somatosensory feedback about the surface properties of the ground. This lack of somatosensory information might be one reason why users of transtibial prosthesis commonly have problems with walking, especially when walking outdoors, ambulating stairs, hills, or on uneven grounds (3–6).

Other serious problems that commonly occur following amputation are phantom limb pain (PLP) and phantom limb sensations that both are felt in the missing part of the limb (7). With about 70% of lower limb amputees, PLP is a rather frequent sequela of amputation (8, 9). PLP might hinder the use of a prosthesis and negatively affect many of subjects’ daily activities (10, 11). PLP often occurs either as constant pain or as pain varying across the day or as separate pain attacks of different intensity and duration (12). As PLP is often unpredictable and strong, it impairs almost all everyday activities and contributes to depression and anxiety (12). Thus, PLP is considered to represent a major burden for most patients following amputation.

A large number of factors have been demonstrated to contribute to the genesis and maintenance of PLP (13). Specifically, PLP is associated with neuronal reorganization in the peripheral somatosensory nervous system and motor system, in the spinal cord, and the central representation areas of the amputated limb and its neighboring areas in the primary sensory and primary motor areas of the brain (14, 15). Peripheral alterations comprise ectopic activity in deafferented nerves and in the dorsal root ganglion, and formation of ephapses and/or neuroma. Spinal changes include reorganization of the body map and sensitization of spinal transmission neurons. Supraspinal changes comprise plastic changes in the sensorimotor nervous system. Specifically, central changes include general disinhibition, unmasking of preexisting connectivity between neurons, sprouting, map remodeling, loss of neurons and neuronal function, denervation, alterations in neural and glial activity, and sensory–motor and/or sensory–sensory incongruence (14).

While cortical reorganization was shown to represent a central key for the development of PLP, the question arose whether a modification of this maladaptive reorganization might lead to a reduction of PLP. Some evidence for this association was provided by a study on amputees who received a functional Sauerbruch arm prosthesis instead of a cosmetic prosthesis. The Sauerbruch prosthesis is a mechanical device connected to the biceps muscle by cables that operate a rod terminating at its proximal end in a surgically created tunnel. Movements of the prosthesis are triggered by contraction causing the fingers to fold to a grip with different force according to the strength of the muscle contraction. Relaxation of that muscle opens the fingers and releases the strength of the grip. Thus, there is direct motor control of and somatosensory feedback from the prosthetic hand originating in the muscles of the stump (16, 17). While the Sauerbruch prosthesis provides feedback from the biceps muscles during grasping, the cosmetic prosthesis does not feedback any activity and sensation of the prosthesis. In a study on effects of the Sauerbruch prosthesis on PLP, we found substantially lower PLP for all users of Sauerbruch prosthesis as compared with the users of a cosmetic arm prosthesis. Thus, we hypothesized that somatosensory feedback of actions with a prosthesis might significantly affect PLP and relief the burdens of amputation. Similarly, Lotze et al. (18) reported that users of a functional myoelectric arm prosthesis exhibited less PLP and less cortical reorganization in the primary somatosensory cortex (SI) than users of a cosmetic arm prosthesis. Besides this, a direct relationship between reduction of PLP and normalization of the amputation-induced reorganization in SI was demonstrated in upper limb amputees using discrimination training (19). These authors trained arm amputees for 2 weeks to discriminate patterns of electrical stimulation at the stump. They found a reduction in PLP that coincided with a reduction of the amputation-induced reorganization in SI. Furthermore, we recently applied the somatosensory activity feedback (SAF) training to a myoelectric arm prosthesis and trained forearm amputees with this SAF prosthesis for 2 weeks. This training resulted in significantly increased functionality of movements with the prosthesis and a reduction of PLP (20).

The incidence of lower limb amputations is higher than that of arm amputations. However, there are only a few studies on the course of prosthesis use and PLP in leg amputees up to now. Especially, a system with somatosensory feedback from the prosthetic foot has not been tested systematically so far. Therefore, the aim of this study was to test whether training with a leg prosthesis with somatosensory feedback affects patients’ PLP and increases the functionality of the prosthesis use in lower leg amputees like in lower arm amputees.

## Materials and Methods

### Subjects

The study includes 14 unilateral lower limb amputees (5 females, mean age = 56.3 years ± 11.6, range: 27–76). Patients were recruited through advertisements and from patient pools of the German Social Accident Insurance (Deutsche Gesetzliche Unfallversicherung, DGUV), a nation-wide insurance system for medical treatment and rehabilitation of injuries and diseases caused at the work place and local dealers of rehabilitation gear. A telephone interview was performed assessing inclusion criteria. These criteria were the presence of a transtibial amputation subsequent to trauma, PLP, and the ability to walk at least 800 m using the leg prosthesis. During this telephone interview, patients were also informed about the study and asked for further contact details. When inclusion criteria were satisfied and patients agreed, patients were offered participation in the study. Characteristics of participating amputees are shown in Table 1. This study was carried out in accordance with the recommendations of Ethics committee of the Friedrich Schiller University Jena with written informed consent from all subjects. All subjects gave written informed consent in accordance with the Declaration of Helsinki. The protocol was approved by the Ethics committee of the Friedrich Schiller University Jena (No. 1312-05/04).

**Table 1:** Demographic and clinical characteristics of patients.

No.	Sex	Age cat.	TSA	Side	Reason	HSQ	LCI basic	LCI advanced	Pain characteristics	Pre PLP	Train PLP
01	M	40–45	215	L	Trauma	10	28	24	Pain attacks, pain-free between	7.80	6.78
02	M	26–30	14	R	Trauma	12	26	28	Pain attacks, pain-free between	0.40	1.11
03	M	50–55	188	R	Trauma	12	28	28	Constant pain with slight variation	NP	NP
04	M	50–55	408	L	Trauma	9	28	19	Pain attacks, pain-free between	3.00	2.67
05	M	60–65	39	R	Trauma	12	28	28	Pain attacks, pain-free between	NP	NP
06	M	60–65	38	R	Inflammation	10	28	21	Pain attacks, pain-free between	2.40	2.30
07	F	50–55	27	L	Trauma	8	28	25	Pain attacks, pain-free between	2.50	1.30
08	M	50–55	146	L	Trauma	12	28	28	Pain attacks and pain between	4.10	3.90
09	F	66–70	517	R	Embolism	9	22	11	Pain attacks, pain-free between	0.80	0.60
10	F	56–60	484	L	Trauma	12	28	28	Pain attacks, pain-free between	2.00	0.63
11	F	50–55	60	L	Trauma	11	28	24	Pain attacks and pain between	1.78	1.00
12	F	76–80	648	R	Trauma	10	28	27	Pain attacks, pain-free between	0.30	0.80
13	M	60–65	32	R	Embolism	12	28	28	Pain attacks, pain-free between	0.00	0.20
14	M	55–60	390	L	Trauma	12	26	25	Pain attacks, pain-free between	2.50	0.90

*Demographic and clinical characteristics before training*.

*M, male; F, female; Age cat., age category in years; TSA, time since amputation in months; Side, side of amputation; R, right; L, left; Reason, reason for amputation; HSQ, sum score of Houghton Score Questionnaire (21) indicating that most patients used their own prosthesis intensively and frequently (maximal possible score: 12); LCI, subscores of Locomotor Capability Index (22) measuring prosthetic mobility (maximal possible score: 28); Pre PLP, averaged numerical rating scale (NRS) (0–10) at evening during the waiting period; Train PLP, averaged NRS (0–10) at evening during the training period; NP, not provided by the patient; PLP, phantom limb pain*.

### Experimental Design

**Figure 1:**
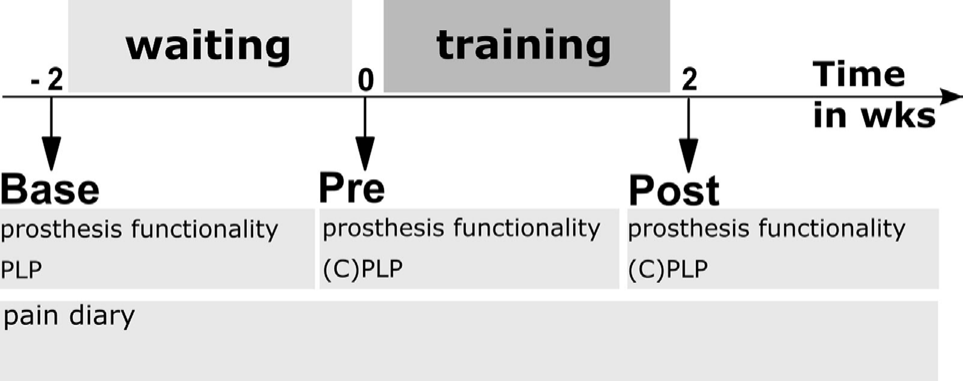
Experimental design. Base—baseline assessment; Pre—assessment directly before first training session; and Post—assessment after last training session. Waiting period—patients used their own cosmetic prosthesis without feedback during everyday life. Training period—patients used their own prosthesis that was equipped with a somatosensory feedback system during 10 days of prosthesis training. Prosthesis functionality was assessed using questionnaires and walking tests, PLP—phantom limb pain characteristics, CPLP—retrospective change of PLP during past 2 weeks.

The study used a within-subjects design. The study included a baseline assessment followed by a 2-week waiting period, a pretraining assessment (Pre), a 2-week training period, and a posttraining assessment (Post) (Figure 1).

Baseline assessment (Base) comprised a series of psychological and psychophysiological tests to describe our subjects with respect to different aspects influencing pain perception and functionality of the prosthesis that was worn by the patient before our training. This includes questionnaires concerning the following:

(a) prosthesis functionality before training [Houghton Score Questionnaire (HSQ) (21), Locomotor Capability Index (LCI) (22), Trinity Amputation and Experience Scales (TAPES) (23), and Amputee Body Image Scale (ABIS) (24)],
(b) phantom characteristics and pain including core dimensions (25) {half-standardized interview adapted from Winter et al. (26), the German Version of the McGill Pain Questionnaire (27), scores on physical functioning according to the German Version of the West Haven-Yale Multidimensional Pain Inventory, MPI-D (28), the German Version of the Pain Catastrophizing Scale (29, 30), scores on emotional functioning: the German Version of the Becks Depression Inventory, BDI-II (31, 32), the State-Trait Anxiety Inventory [STAI-G (33)], and the German version of the Health Survey [SF-36 (34)]}, and
(c) the assessment of brain functioning using functional magnetic resonance imaging and magnetoencephalography when possible. FMRI and MEG data are not addressed in this manuscript and will be presented elsewhere.

After baseline, patients started filling in a pain diary during the 2-week waiting period. Patients were asked to note their current PLP and stump pain on a numerical rating scale (NRS) ranging from “0” (no pain) to “10” (pain as bad as it ever could be) three times per day between baseline and post assessment. Patients were further asked to note each day how many hours they wore the prosthesis. In addition, medication and sleep disturbances were to be noted as well.

After the waiting period, the 2-week training period started with a pretraining assessment (Pre) comprising an evaluation of phantom characteristics and pain similar to the baseline with additional items on the variability of the intensity and frequency of PLP (CPLP, see Section “Assessment of Pain” for details) and functionality of the prosthesis use (question electrocutaneous feedback, Q_EF, see Section “Assessment of Prosthesis Functionality” for details). Furthermore, the goals for the training were defined using a goal attainment scale [GAS (35), see Section “Assessment of Prosthesis Functionality” for details]. In addition, an obstacle course [similar to Ref. (36)] and a 2-Minute Walk Test (37) were performed. Thereafter, patients took part in a daily prosthesis training for 10 days (Figure 1) (38). There were no limitations on other treatments or medications during the study. At the first training day, somatosensory discrimination of electrical stimulation was trained; discrimination was assessed before and after somatosensory discrimination training. The standard training starting with the first day is described in Section “Training” in detail.

At the last training day, we performed a similar assessment as before the training including the evaluation of phantom characteristics and pain, the functionality of the prosthesis, the assessment of goal attainment (GAS) evaluated by trainer and patient, the completion of the obstacle course, and the 2-Minute Walk Test. We also performed a half-standardized interview on the usability of SAF prosthesis, training, and asked for ideas to further improve training and prosthesis in future.

### Assessment of Pain

Characteristics of pain were assessed by a pain diary during the waiting and training periods. From Base to Post, participants kept a pain diary to assess their current PLP three times a day (morning, noon, and evening) using an 11 points NRS with the end points 0 = “no pain” and 10 = “strongest pain.” NRS is considered a valid and reliable tool for measurement of pain intensity (25). The average of these three assessments provided a daily mean PLP score. In addition, there were retrospective assessments of changes of PLP intensity and frequency during the waiting period (the assessment took part immediately before training, Pre). Changes of PLP intensity and frequency (CPLP) during the training period were additionally assessed at Post using a visual analog scale (10 cm) with two poles, i.e., “strongly reduced” and “strongly increased,” and “no change” in the middle of the line.

### Assessment of Prosthesis Functionality

#### Handling of the Feedback System

To assess whether patients could use the feedback, the discrimination performance and handling of the prosthesis were assessed. 1. Discrimination performance was assessed twice, once before patients learned to discriminate the three possible stimulation patterns and once after the learning session on the first training day. Two electrodes were mounted on the residual limb and the subjects were tasked with identifying when the lower, upper, or both were active. Each test comprised a random presentation of 25 stimulus patterns of these three possibilities (lower, upper, and both electrodes).

Discrimination performance was calculated as percent correct discriminations. 2. Patients were requested to provide ratings on a 5-point Likert scale ranging from 1 (“appropriate”) to 5 (“not appropriate at all”) at the first and at the last day of training in response to the following statement: “I can interpret and evaluate the electrocutaneous feedback very well” (Q_EF).

#### Performance in Target Activities (GAS)

Before the first training day, patients and trainer negotiated personal target motor tasks that patients aimed to accomplish until the end of the training period. Tasks included, for example, using the prosthesis for walking on soft and bumpy grounds or safely walking uphill and downhill (35, 38, 39). After the end of the training, patients and trainers rated the achievement of each goal with 1—deteriorated, 2—maintained initial state, 3—goal 25% attained, 4—goal 50% attained, 5—goal 75% attained, 6—goal 100% attained.

#### Performance in Standardized Activities

##### Obstacle Course

The ability to navigate uneven terrain was assessed on a standardized, 88-m obstacle course that included wood chips, little blocks of wood, pea gravel, coarse gravel, walking on a gym mat, as well as a cobblestone ramp and stairs. Subjects were asked to walk at a self-determined walking speed while overall time was measured (36) at Pre and Post. Training on the obstacle course was not part of the training sessions, hence, if the walking test after the training period was accomplished significantly faster than at the beginning of the training period, then it was considered a training effect.

##### 2-Minute Walk Test

This test was administered at Pre and Post. The test was performed in a quiet uncarpeted corridor. There were two pylons in a distance of 25 m. Subjects were asked to walk as far as they could around the pylons in 2 min without any further encouragement. The test administrator walked behind the subject to minimize the effect of pacing. Subjects were provided with clear instructions and were allowed to rest during the 2-min time period, if required. Distance walked was recorded in meters.

#### Interview

At Post, subjects were interviewed about the usability of the prosthesis with and without feedback and asked to specify which one of both they prefer in the future and to explain why they prefer it using their own words.

### Technical System

**Figure 2:**
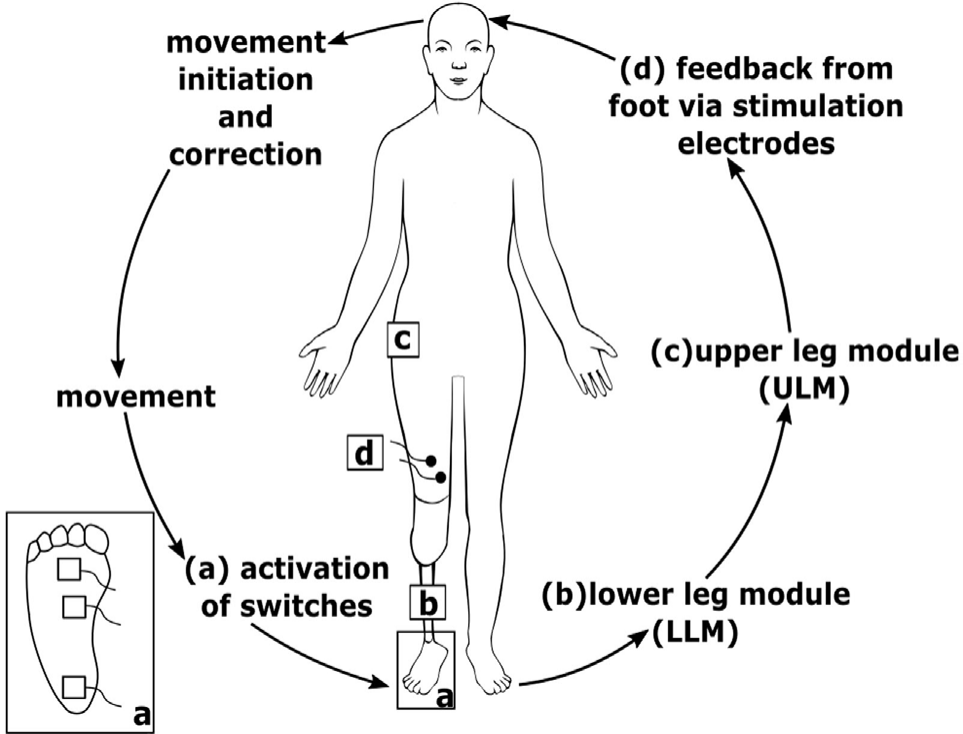
Scheme of the technical system. (a) Sensors at the prosthesis foot detect ground contact and send signal to lower leg module (LLM) (b); LLM (b) sends information to upper leg module (ULM) (c) *via* Bluetooth connection; ULM (c) generates electrocutaneous stimulation signals that are applied *via* stimulation electrodes (d) at the thigh; inset (a) bottom view of the prosthesis foot with three sensors.

Participants used their own cosmetic lower limb prosthesis, which was technically adapted to include a somatosensory feedback system (see Figure 2). We developed an add-on feedback kit that allowed a fast and sensitive response while walking on bumpy grounds, walking curb stone edges and cobbled pavements, stairs, and skewed planes.

**Figure 3:**
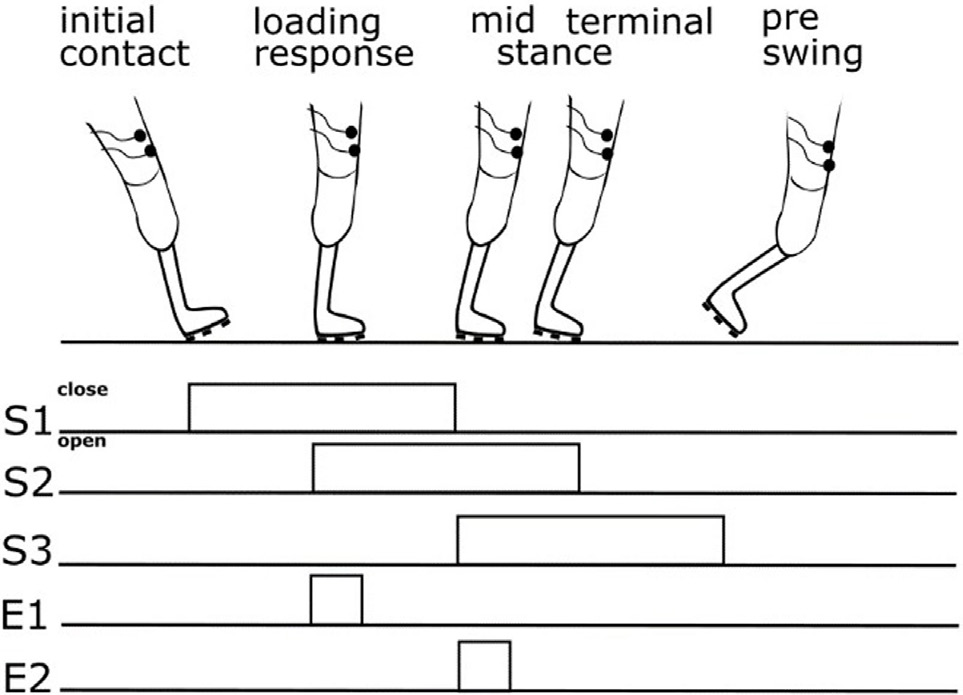
Scheme showing relation between gait cycle phase, switch closure, and feedback. S1—switch at heel, S2—switch at middle foot, S3—switch at bunion, E1—upper electrode signaling closure of the switch at the middle foot, and E2—lower electrode signaling closure of the switch at bunion.

The somatosensory feedback kit includes three pressure sensors/switches fixed to the sole of the prosthesis foot (heel, middle outer surface, and bunion) at the load line of the prosthesis foot (38). The load line was assessed using a standardized foot pressure measurement system (medilogic, Schönefeld, Germany). Switch closures were registered by a lower leg module (Figure 2) and sent *via* Bluetooth connection to an upper leg module (ULM). The ULM generated electrocutaneous stimulus patterns delivered to the stump. The ULM including the electrical generator can be bonded to the belt. Electrocutaneous stimulation at the stump comprised a 77 Hz rectangular stimulus pattern of 12.9 ms duration with an intensity that produced a clearly perceivable, but non-painful stimulus (max output: 64 mA at 25 V). We decided to give very simple SAF. We assumed and confirmed by asking the patients that the contact to ground at heel is sufficiently recognized by the patients *via* proprioceptive feedback of the stump in the shaft. However, we supposed that further rolling off the foot is not as clear as heel contact. Therefore, we aimed at signaling a contact of the middle of the foot and of the bunion (see Figure 3). To avoid somatosensory overload, signals from the switches of middle foot and bunion were only allowed to activate the electrode if they appeared after closure of the switch at the heel. This avoids continuous stimulation during standing. The stimulation pattern itself remained unchanged, however, as switch closure differed on different ground conditions, patterns of switch closure changed. This allows to detect edges and borders at the foot, twisting, and tilting, etc.

The procedure for applying electrocutaneous feedback has already been described in detail elsewhere (40, 41). In our study, SAF was provided as the closure of switches *via* two adhesive surface Ag/AgCl electrodes (50 mm; spes medica, Genova, Italy).

### Training

The whole training comprised 10 days (10 working days) offered across a period of 2 weeks. Each training day included two training sessions of approximately 2 h that were separated by a break of 30–60 min (38).

At the beginning of each training day, electrodes for electrocutaneous SAF were attached to the residual leg in the middle of the thigh above the liner under an angle of 45° with respect to femur. This was done to increase the possibility to discriminate the stimulations spatially and to rebuilt an image of the foot on the thigh (bunion down). Stimulation intensity at each electrode was tuned to secure a clearly non-painful percept. Finally, the system was checked for correct work. Using a surgical crayon, the position of electrodes was marked at the first training day to ensure that the position of the electrodes remained the same between the training days.

As discrimination of electrical stimuli to the stump is an indispensable prerequisite for the proper function of the prosthesis with SAF (*via* the electrical stimulation), stimulation and discrimination abilities of each patient were tested in advance during the first training day. Participants were familiarized with this stimulation and learned to discriminate three possible stimulation patterns (upper electrode, lower electrode, and both electrodes). This discrimination was quite easy so that all patients learned to discriminate these three stimulation possibilities within 30 min.

Then, each training session started with warming-up exercises of approximately 30 min where patients walked on a treadmill or played balance and step games using a commercial video game console. Thereafter, training started outdoors by walking on sidewalks to near downtown goals or on park or forest paths with different ground surfaces. Several therapy principles were borrowed from the group’s expertise with constraint-induced movement therapy (42–47). So ground surfaces were chosen individually for each patient according to the actual walking capacities and the overall goals expressed in the GAS at pre training. Care was taken to neither overstrain or under-challenge each patient. When progress in walking became obvious to trainer and patient within a training session, the difficulty of ground surfaces and the length of single walks were increased in consultation with the patient. The second session per day mainly contained the same sequence of walking conditions. The trainer logged type and duration of walking tasks as well as positive and adverse events.

### Statistical Analysis

With respect to the aims of the study, we chose the following primary endpoints: (a1) manageability of the feedback system as measured *via* Q_EF and increase of discrimination performance, (a2) improvement of desired motor activities of the patient as measured *via* GAS, (a3) functional improvement in standardized activities as measured *via* performance in standard tests, (a4) reduction of current PLP intensity as measured by the NRS of a pain diary, and (a5) personal impression of change of PLP (CPLP). Regarding the CPLP, the retrospective assessment during the training period was compared with the retrospective assessment during waiting period. Normal distribution of data was assessed using the Shapiro–Wilk test. If data were normally distributed, then *t*-tests for dependent samples were used. Wilcoxon signed rank tests were used when data were not normal distributed. Significance level was set to 5%. Data were analyzed with IBM SPSS Statistics 24 (IBM Corporation, NY, USA). Treatment was considered effective according to the consistency principle (48–50) which implies that no adjustment for multiple endpoints will be necessary, if statistical significance is demonstrated at a prespecified nominal level for the majority of primary endpoints. As this is a preclinical study, we also report the qualitative data that were gathered on functionality of the prosthesis in the interview after the training, and we report adverse events that were spontaneously reported during the training period.

## Results

### Prosthesis Functionality

#### Handling of the Feedback System

There was a significant increase of discrimination performance during discrimination training compared with testing before the first training session (see Table 2). Furthermore, patients learned to interpret the sensory feedback. Patients answered to this item at the first assessment on average with “neither applicable nor not applicable” (M = 2.6), whereas patients rated at the last assessment “rather applicable” (Table 2).

**Table 2:** Summary of major statistical results.

Measure	Pre		Post		*t*/*Z*	Df/*n*	*p* (One sided)
	M	SD	M	SD			
**Section 1: prosthesis functionality**							
Somatosensory discrimination performance (in %)	52.57	35.29	77.14	24.28	3.18	13	0.007
Interpretation of feedback	2.6	1.3	3.8	1.31	3.19	13	0.007
Goal attainment scale (GAS)/target activities/patient^a^	n.a.	n.a.	3.8	1.1	5.9	13	0.0001
GAS/target activities/trainer^a^	n.a.	n.a.	3.9	1.1	6.75	13	0.0001
Obstacle course (in s)	117.8	51.62	108.3	43.25	−3.3	14	0.001
2MWT (in m)	135.7	24.7	139	25.1	1.54	13	0.07

**Section 2: pain**							
Pain diary (evening)	2.3	2.12	1.9	1.9	−2.09	11	0.03
CPLP intensity^a^	3.9	14.02	−22.05	41.44	−1.78	13	0.038
CPLP frequency^a^	3.72	13.42	−21.72	44.4	−1.997	13	0.023

*Pre, assessment before training which was after waiting period; post, assessment after training; M, mean; n.a., not applicable*.

*^a^Retrospectively assessed for waiting period (Pre) and/or training period (Post), Df/*n*—degrees of freedom (*t*-test), or number of subjects (*Z*-test)*.

#### Performance in Target Activities (GAS)

Functionality in personalized everyday goals increased according to the judgments of both patients and therapists. Achievement of everyday goals during training was rated at the last day on average as “50% achieved” (Table 2). Patients were allowed to name up to five everyday goals. Everyday goals for nearly all patients comprised secure ambulation on soft and bumpy grounds, such as grass, off-road, cobbled streets, gravel, and slippery ground. Typical goals were also improved reaction during ambulating unexpected obstacles, enlarge the limits of movement (22 times named), walking longer distances, more efficiently, less energy-sapping (10 times named), mastering stairs without handrail and with changeover step (5 times named), improving gait (7 times named), and walking without support (3 times named). Other everyday goals were jumping with both legs, walking without visual control, mastering ramps, increasing flexibility, and balance.

#### Performance in Standardized Activities

Patients mastered the obstacle course faster after the training period than before the training period (see Table 2). The distance they walked at normal pace in the 2-Minute Walk Test was not significantly increased after the training compared with before the training (see Table 2).

#### Interview

Patients reported that they or their partner had noticed improvements of movement/gait (5/14). With SAF prosthesis, one patient with reduced telescoping felt that his stump was at “normal length” again. Two participants reported on longer power of endurance during walking. 9/14 patients preferred the SAF prosthesis over their own prosthesis without SAF due to the following reasons: wanted to continue using the SAF prosthesis (3×), longer endurance during walking without breaks with SAF (1×), SAF helps against PLP (3×), walking in the forest is easier with SAF prosthesis (1×). One person did not name any reason. Five patients preferred their own prosthesis. Reasons were that cable and upper limb module perturbed (3×), feedback perturbed (1×), and/or they felt more fit with their own prosthesis (3×).

### Pain

#### Pain Diary

**Figure 4:**
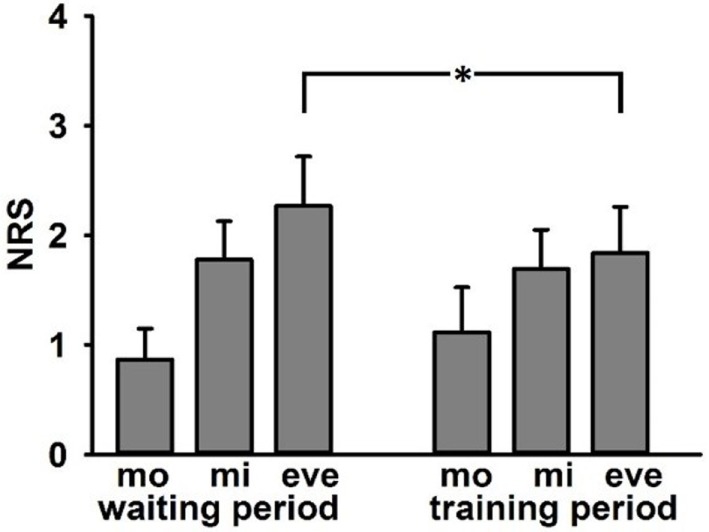
Adjusted mean values (±SE) of ratings of current phantom limb pain in numerical rating scale in pain diary (0 = no pain, 10 = strongest pain) separated for waiting and training period—mo, morning; mi, midday; and eve, evening. Asterisk indicates significant differences in *post hoc* tests.

12 of 14 patients completed pain diaries thoroughly. Mean scores were entered into a repeated measurements ANOVA with the factors Time of day (3-levels: morning, midday, evening) and Period (2 levels: waiting period and training period). There was a significant main effect of Time of day *F*(2/10) = 5.06, *p* = 0.03 with lowest values during morning and highest values for evening. Importantly, there was a significant interaction effect Time of day*Period *F*(2/10) = 5.55, *p* = 0.024. *Post hoc* tests revealed lower mean values in the evening of the training period (M = 1.8, SD = 1.9) than in the evening of the waiting period (see Figure 4).

#### Retrospective Evaluation of Pain (CPLP)

8/14 participants reported that PLP had changed during training period whereas only 1/14 had reported change of PLP after waiting period [*t*(13) = −2.45; *p* = 0.007]. Retrospective PLP intensity reduction was significant for training period vs. waiting period (see Table 2). Retrospective PLP frequency was also significantly reduced for training period vs. waiting period (see Table 2). One patient reported an increase of PLP during waiting period, and one patient reported an increase of PLP during training period. No patient reported a change of quality after waiting period. Three patients reported that quality of PLP had changed during training period (“burning has become warmth,” “stabbing, cutting pain became pulsating,” “dull, less feeling of a phantom limb”). Seven patients reported changed phantom sensations after training period (“phantom limb appears longer”; “less frequent feeling of phantom foot tangling from knee joint,” “more frequent phantom sensations in heel and leg,” “permanent prickling,” “pressure, squeezing now numb,” “soft prickling,” “less frequent, intensity similar to healthy foot and lower leg,” “different temperature and position of phantom limb,” “less frequent,” “less frequent and less intense,” “no phantom sensations anymore”).

#### Adverse Events

The trainer documented adverse events that were spontaneously reported by patients. During training period, the following adverse events occurred: strain-induced stump pain, blisters or redness at the stump (4×), sudden PLP, and difficulties with prosthesis fit because of sweat and gliding in the shaft. Complaints were transient. The patients already knew the complaints from intensive usage of their own prosthesis in everyday life. One participant regularly reported stump pain during walking with the prosthesis after about 40 min. of training. The stump pain led to increased frequency and intensity of PLP at the same day. In addition, this participant showed increased sweating at the residual limb, which prevented adhesion of feedback electrodes. As the training with such an SAF prosthesis comprises an intensive walking load, an optimal prosthesis fit at the stump is a necessary prerequisite for the intervention.

#### Exploratory Analyses for Adherence With IMMPACT Recommendations (25)

Emotional functioning as assessed by the sum scores of the BDI-II (31, 32) was not significantly reduced at Post compared with Pre [M_pre_ = 6.69, SD = 7.5; M_post_ = 5.69, SD = 5.91, *t*(12) = −1.01, *p* = 0.15, one sided]. Physical functioning as assessed by the German Version of the West Haven-Yale Multidimensional Pain Inventory, MPI-D (28) did not differ significantly between Pre and Post [M_pre_ = 1.5, SD = 1.48, M_post_ = 1.09, SD = 1.01, *Z* = −1.29, *p* = 0.098, one sided].

## Discussion

The study shows in unilateral transtibial amputees that training with an SAF system providing electrocutaneous feedback to the thigh during walking reduces PLP and improves functionality of movements with the prosthesis considerably for some patients.

Specifically, there was a significant interaction between Time of day (morning, midday, and evening) and Period (waiting vs. training period). Patients reported lower PLP at the end of the day in the training period, which was the time, when patients reported strongest PLP intensities in the waiting period. Similarly, most patients reported a reduction of intensity and frequency of PLP during training but not during waiting period before training. This decrease of PLP is in line with our former results on patients with upper limb amputation (20, 51). Hence, the use of prostheses with somatosensory feedback is an option to reduce PLP not only in upper limb amputees, but also in lower limb amputees. In addition, this result is in accordance with the analgesic effects of somatosensory discrimination training (19), mental imagery (52), graded motor imagery (53–55), mirror therapy (56, 57), or phantom motor execution, facilitated by myoelectric pattern recognition and virtual reality (58, 59). The result is extending the knowledge about the relation between prosthesis usage and pain. Formerly, PLP was associated with a decreased use of a prosthesis (2, 11). As our studies show the decreased use of prostheses because of PLP might in part be counteracted by adding somatosensory feedback to the prostheses (13, 17, 20, 51). Moreover, even when amputees use a standard prosthesis frequently, the add-on of SAF reduces PLP.

As a second important result, patients reported more stable, better control of walking, especially on bumpy and soft grounds, as well as on larger walking distances. Similarly, improved functionality of movement was apparent in the shorter time needed to master an obstacle course after the training. Importantly, the study shows that somatosensory feedback specifically improves functionality in usually difficult situations for transtibial amputees such as walking uphill and downhill, walking on uneven ground and ambulating stairs (3, 4). The prosthesis functionality scores in the LCI and HSQ that were obtained before training indicate that our sample did start the training with already good functionality of movement. Besides such good baseline conditions, there was still a need to improve functionality, and this need was achieved by the training. This indicates that somatosensory feedback increased the functionality of movement with the prosthesis specifically to improve everyday life. It eases the usage of prostheses in daily life, and this might increase acceptance and satisfaction with the prosthesis (11). Satisfaction with the prosthesis is an important issue as it contributes to a successful rehabilitation after amputation. Studies in arm amputees suggest that one reason for reduced satisfaction with a prosthesis was missing feedback from the prosthesis (60). Furthermore, most arm amputees have to compensate this loss by increasing visual control of the prosthesis (61–63). To the best of our knowledge, there has been no similar study in lower limb amputees asking for patients’ wishes concerning prosthesis functionality. One important point for patients is feedback information about the missing limb from the prosthesis. Such information is not provided by most commercial lower limb prostheses. Our study shows that somatosensory feedback information does indeed have the capacity to improve functionality of movement in everyday life.

The beneficial effects of feedback on functionality of movement and reduction of PLP are in line with newer developments of hand prostheses like osseo-integrated prostheses (64) and bidirectional hand prostheses (65, 66). Such prostheses successfully use direct nerve stimulation for the control of the prosthesis; however, until now, it has been only used in single cases. Different to such approaches, an SAF system as described in this study provides a simple to use, low cost technique for leg amputees who are already equipped with a prosthesis and who are not willing to undergo surgical procedures. The usability of the add-on feedback system is supported by the answers of the majority of patients who reported that they would like to continue the usage of the somatosensory feedback prosthesis immediately in everyday life.

The precise mechanisms that underlay the beneficial effects of feedback prostheses are not completely known yet. With respect to central factors that contribute to PLP, both the functionality of a limb and pain in a limb are reflected in the organization of the primary somatosensory cortex (SI) (67, 68). Specifically, PLP is associated with reorganization of areas neighboring the deafferented representation (69, 70) and disturbed organization in the representation of the amputated extremity (71). Our hypothesis for the use of SAF prosthesis is based on postulates that additional and meaningful information from the prosthetic hand or from the prosthetic foot that is applied to body parts near to the amputation line (stump) might result in a reduction of reorganization and consequently to a reduction of PLP. We recently found that the therapy with SAF prosthesis in arm amputees changed the cortical thickness in small brain areas in the visual stream and the post-central gyrus ipsilateral to the amputation (72). While this result points to a possible importance of the visual stream, further research is needed to identify underlying mechanisms and their relative contribution for PLP reduction when SAF prostheses are used.

The study provides a proof of concept that SAF prostheses have beneficial effects on PLP and functionality in lower limb amputees. To further validate the results reported here, it is essential to replicate this result in a larger sample of lower limb amputees. As the technology of prostheses for daily use for lower limb amputees has not changed dramatically since 1996, the need for such technology still represents an important issue for most amputees with prostheses (73).

Future studies need to show to which population of amputees the effect can be generalized. One patient in our study reported strain-induced residual limb pain that was accompanied by PLP after some time. The sweating that was associated with the pain hindered the adherence of the electrodes and, therefore, counteracted the feedback system. This shows that a good prosthesis fit is a necessary prerequisite for such a therapy. As there are many mechanisms contributing to PLP, there might be some patients who will benefit more from such a therapy than others.

Furthermore, although the majority of patients preferred the somatosensory feedback prosthesis, there were still four patients, who were perturbed by the design of the SAF system, and one patient, who was perturbed by the SAF itself. Future research could aim at even better designs and more natural feedback (e.g., vibratory or tactile). Furthermore, it seems necessary to shape the requirements for applying SAF at the thigh. Moreover, it is also possible that patients improved in function as a result of dedicated one-on-one training with a therapist for ten consecutive days. We cannot exclude that the effects are not simply a result of the training having the effect of physical therapy or athletic training or, at least, mediated by these factors. Besides that, the therapist actively shaped the behavior of the patient during the various walking tasks and was not only simply there to observe and for safety. However, we believe that most of our effects are directly linked to the effect of the additional somatosensory feedback as most of the patients had extensive therapy after the amputation and that therapy was most often on a one-by-one basis with an experienced therapist.

An open issue with respect to functional arm prostheses is the discussion whether restoring the somatosensory function or restoring the motor function of the affected limb is more efficient for the improvement of functionality and the reduction of PLP. In arm amputees, such an answer is difficult as functional SAF prostheses restore functionality of movement (i.e., grasping) and provide somatosensory feedback at the same time. Training with the SAF prosthesis in lower limb amputees does more selectively improve somatosensory functions while the motor control of lower limb prostheses remains limited. Therefore, our study in lower limb amputees gives a hint that SAF itself might be an important component for the beneficial effects with SAF prostheses on functionality and pain.

In summary, our study of lower leg amputees trained on a prosthesis with somatosensory feedback from the sole of the prosthetic foot demonstrates a remarkable reduction of PLP. Therefore, we suggest the use of such a prosthesis as a therapeutic opportunity to reduce PLP in lower limb amputees.

## Ethics Statement

This study was carried out in accordance with the recommendations of Ethics committee of the Friedrich Schiller University Jena with written informed consent from all subjects. All subjects gave written informed consent in accordance with the Declaration of Helsinki. The protocol was approved by the Ethics committee of the Friedrich Schiller University Jena (No. 1312-05/04).

## Author Contributions

TW, WM, and GH conceived the investigation. CD, KB, SN, TW, and WM designed the experiment. CD, KB, SN, and SS collected data. CD analyzed data and wrote the first draft. All the authors revised the draft critically for important intellectual content and agreed to be accountable for the content of the work.

## Conflict of Interest Statement

All authors declare that the research was conducted in the absence of any commercial or financial relationships that could be construed as a potential conflict of interest.
